# Nanoparticulate Radiolabelled Quinolines Detect Amyloid Plaques in Mouse Models of Alzheimer's Disease

**DOI:** 10.4061/2009/481031

**Published:** 2010-01-27

**Authors:** Celeste A. Roney, Veera Arora, Padmakar V. Kulkarni, Peter P. Antich, Frederick J. Bonte

**Affiliations:** ^1^Division of Radiological Sciences, Department of Radiology, University of Texas Southwestern Medical Center at Dallas, Dallas, TX 75390, USA; ^2^The Nuclear Medicine Center, University of Texas Southwestern Medical Center at Dallas, Dallas, TX 75390, USA

## Abstract

Detecting aggregated amyloid peptides (A*β* plaques) presents targets for developing biomarkers of Alzheimer's disease (AD). Polymeric n-butyl-2-cyanoacrylate (PBCA) nanoparticles (NPs) were encapsulated with radiolabelled amyloid affinity ^125^I-clioquinol (CQ, 5-chloro-7-iodo-8-hydroxyquinoline) as in vivo probes. ^125^I-CQ-PBCA NPs crossed the BBB (2.3 ± 0.9 ID/g) (*P* < .05) in the WT mouse (*N* = 210), compared to ^125^I-CQ (1.0 ± 0.4 ID/g). ^125^I-CQ-PBCA NP brain uptake increased in AD transgenic mice (APP/PS1) versus WT (*N* = 38; 2.54 × 10^5^ ± 5.31 × 10^4^ DLU/mm^2^; versus 1.98 × 10^5^ ± 2.22 × 10^4^ DLU/mm^2^) and in APP/PS1/Tau. Brain increases were in mice intracranially injected with aggregated A*β*_42_ peptide (*N* = 17; 7.19 × 10^5^ ± 1.25 × 10^5^ DLU/mm^2^), versus WT (6.07 × 10^5^ ± 7.47 × 10^4^ DLU/mm^2^). Storage phosphor imaging and histopathological staining of the plaques, Fe^2+^ and Cu^2+^, validated results. ^125^I-CQ-PBCA NPs have specificity for A*β* in vitro and in vivo and are promising as in vivo SPECT (^123^I), or PET (^124^I) amyloid imaging agents.

## 1. Introduction

Alzheimer's disease (AD), a neurodegenerative disorder principally of the elderly, is the most prevalent form of dementia. The cognitive decline associated with AD drastically affects the social and behavioral skills of patients living with this disease. Notwithstanding the social impact, AD, also places great financial burdens on patients, families, and the community as a whole. Furthermore, therapeutic strategies to probe the central nervous system (CNS) are limited by the restrictive tight junctions at the endothelial cells of the blood brain barrier (BBB). To overcome the impositions of the BBB, polymeric biocompatible drug carriers have been applied to the CNS for many applications, including cancers; however, the field of drug carrier technology is not well developed in AD research [1]. Polymeric nanoparticles are promising candidates in the investigation of AD since they are capable of opening tight junctions [2] crossing the BBB [3], high drug loading capacities, and targeting the mutant proteins of Alzheimer's [4, 5]. 

Markedly elevated concentrations of zinc, copper, and iron in A*β* deposits of the AD brain are well documented in the literature [6, 7]. Cherny et al. have employed the antibiotic and Cu/Zn-selective metal chelator, CQ, to inhibit A*β* accumulations in AD APP2576 transgenic (Tg) mice. Oral treatments with CQ reduced A*β* deposition by ≈49% and produced no neurotoxicity in a blinded study. The authors [8] speculate that CQ's action on the peptide may facilitate H_2_O_2_ inhibition [8]. Moreover, the authors validated the selectivity of CQ since systemic metal depletion was not found [8]; CQ does not deplete brain tissue of metals, but rather binds to the A*β*-metal complex itself. 

In in vivo experiments with nontransgenic animals treated with CQ, Yassin et al. confirmed significant decreases in the cerebral concentrations of Cu, Zn, and Fe metal ions [9]. However, in Cherny's studies of APP2576 Tg mice, the inhibition of A*β* deposition by CQ caused significant *increases* in the cerebral concentrations of Cu and Zn [8]. This is because APP transgene expression reduces Cu and Zn levels in vivo, while the A*β* concentration steadily rises [10]. There are a few possibilities for the decrease. For example, the metal ions may be effluxed by A*β*, or APP/A*β* may prevent reuptake of the ions [11]. Despite the mechanism, CQ prevents uptake; of the metals by the protein. This action affords metallic access to peripheral brain tissue, and the metal ion concentration increases. Since AD is a syndrome of metal dyshomeostasis, CQ may be able to restore the metal metabolism to its normal state. 

Opazo et al. [12] have explored the A*β*-plaque binding properties of radioiodinated CQ in APP2576 Tg mice ([^125^I]CQ; higher brain retention compared with controls on autoradiography), and in AD patients ([^123^I]CQ; more rapid uptake compared with age-matched healthy controls). SPECT imaging was limited due to low tracer uptake, however, CQ was shown to localize with A*β* pathology [12]. Notably, the authors [12] synthetically precipitated A*β*_1–40_ and A*β*_1–42_ by in vitro methods. The synthetic *β*-amyloid demonstrated ^125^ICQ saturation, which was directed by Zn^2+^; the ^125^ICQ binding was displaced by the heavy metals Zn^2+^ and Cu^2+^, the metal chelator DTPA, and the amyloid affinity dye Congo red. Enhancement of Zn^2+^-^125^ICQ was also shown in concentrated fractions of A*β* from postmortem AD brain. These results validate the effectiveness of CQ as a *β*-amyloid detection agent, and most importantly, correlate literature evidence linking A*β* deposition in AD and cerebral heavy metals [5, 8].

Our preliminary studies with ^125^ICQ showed that the agent crossed the BBB, but was retained too briefly for effective chelation. Therefore, a drug carrier is required to improve the extravascular retention of ^125^ICQ. Polymeric butylcyanoacrylate (PBCA) nanoparticles (NPs) were chosen as the drug carrier. Here, we report that ^125^I-CQ-BCA NPs act as targeted drug carriers with an affinity for amyloid plaques. ^125^I-CQ-PBCA NPs are promising candidates for in vivo brain imaging of the amyloid plaques.

## 2. Methods

### 2.1. Radioiodination of Clioquinol

CQ was radiolabelled with ^125^I (Perkin Elmer, Waltham, MA) by the Chloramine- T (CT) method of radioiodination. Purification of ^125^I-CQ was performed by solvent extraction in dichloromethane (DCM) and H_2_O. The organic layer (e.g., DCM) was evaporated under N_2_ gas, and the ^125^I-CQ was dissolved in dimethylsulfoxide (DMSO, 1 mL). The purification of ^125^I-CQ was analyzed by thin layer radiochromatography (RTLC). The specific purity was >95%.

### 2.2. Synthesis of Polymeric Nanoparticles

The NPs were polymerized as per the modified procedure of Kreuter et al. [13]. A polymerization medium was prepared containing Dextran 70 000 and Tween-80 (polysorbate 80) (both at a concentration of 1% each in 0.1 N HCl) (Sigma, USA); 5.05 × 10^6^ Bq ^125^I-CQ was added to the solution just prior to the addition of butylcyanoacrylate (BCA) monomer. A 1% (w/v) butylcyanoacrylate solution (Sichelwerke, Hannover, Germany) was added drop wise during constant magnetic stirring at 400 rpm. After 3 hours of polymerization, the NP suspension was neutralized with 0.1 N NaOH to complete the polymerization. This solution was filtered with a 0.2 *μ*m filter and purified by ultracentrifugation (Beckman Coulter, Fullerton, CA; 45 K rpm, 1h). The pellet was washed and redispersed in water, which contained 1% Tween-80. The PBCA nanoparticles were then overcoated with 1% Tween-80 by stirring for 30 minutes in phosphate buffer solution (1X PBS), just before in vivo administration. Nanoparticle size (45 nm) was determined by a Zetasizer 3000 HS (Malvern, Worcestershire, UK). 

### 2.3. In Vitro Labeling of Amyloid Plaques

Cortical frontal AD and control brain tissue were obtained from the tissue bank, as approved by our Institutional Review Board (800 *μ*L buffer solution of 0.1% FBS) in the presence of ^125^I-CQ (1.17 × 10^4^ Bq in 100 *μ*L PBS). Brain homogenates were microcentrifuged (13 K RPM, 15 minutes) and the percent binding was calculated. The experimental results provided evidence of preferential binding by ^125^I-CQ to the AD brain tissue, as compared to cortical control brain tissue. 

### 2.4. Aggregation of the A*β*_42_ Peptide

Amyloid protein (1–42), *A* *β*_42_, (0.5 mg) was purchased from Bachem California (Torrance, CA), and dissolved in 1.15 mL PBS (pH 7.4) to a final concentration of 435 *μ*g/mL (100 *μ*M) by magnetically stirring the solution in a closed vessel at 1200 RPM for 7 days at room temperature [14]. After 7 days, the aggregated peptide suspension was visibly cloudy. The aggregated A*β*_42_ was stored in 100 *μ*L aliquots at −20°C until use.

### 2.5. Intrahippocampal Sterotaxic Injection of Aggregated A*β*_42_

All use of animals was in compliance with the regulations of the Animal Resources Center (ARC) of UT Southwestern Medical Center, and approved by the Institutional Review Board (IRB). All mice were anesthetized intraperitoneal (IP) with 100–150 *μ*L of Ketamine HCL (Sigma, St. Louis, Mo). Wild type BALB/C mice (Charles River Laboratories, Wilmington, MA; *N* = 17) were injected by direct stereotaxis (Model 900 Small Animal Stereotaxic Instrument, David Kopf Instruments, Tujunga, CA) with the aggregated A*β*_42_ peptide at a concentration of 1 *μ*g/1 *μ*L, and at a constant flow rate of 60 seconds. A lubricant was placed into the eyes of the mice to prevent over-drying during the experiments. The mice received unilateral injections of either saline or the A*β* peptide aggregate. The injection location corresponded to the mouse brain hippocampus structure CA1, at coordinates −1.5, −1.0, and −1.8, relative to Bregma [15, 16]. The peptide was allowed to grow for 7 days, during and after which time cognitive tests for behavior (Y-maze) were performed. 

### 2.6. In Vivo Imaging of the Plaques by Nanoparticle Administration

The ^125^I-CQ-BCA NPs (1–3 mg) were administered to AD mouse transgenic models by lateral tail vein injection. The A*β*_42_ mice received ^125^I-CQ-BCA NPs at 8 days postinjection of the peptide.

In vivo Storage Phosphor autoradiography (Perkin Elmer Cyclone Storage Phosphor Imaging System; OptiQuant Imaging Software) was used to determine the relative qualitative differences in the brain uptakes of ^125^I-CQ by the AD mouse models and wildtype control mice. Briefly, each mouse was anesthetized I.P. with 100–150 *μ*L of Ketamine HCl (Sigma Aldrich, USA) throughout the duration (5–90 minutes) of the imaging experiment. Postinjection of the radiotracer, the mouse was placed on the phosphor screen with a lead sheet between the film and the animal's body in such a way that the only exposed body part was the head. In this way, background radiation from the body was minimized, and the activity source projected onto the film was from the animal's head region only. Regions of interest (ROI) were drawn in the brain space to obtain information in semiquantitative units (Dynamic Light Units, DLU) per volume space (DLU/mm^2^).

At the conclusion of the imaging experiments, the mice were sacrificed by cardiac perfusion through the left ventricle with 4% paraformaldehyde. The brains were harvested and stored in formalin, before being embedded in wax, and sectioned at a 5 *μ*m slice thickness. The slices were stained with Congo red (amyloid plaques), Prussian blue (Fe^2+^), and Rubeanic acid (Cu^2+^).

### 2.7. AD Transgenic Mice

Mice with a double mutation (APP/PS1) for Alzheimer's disease were commercially purchased (*N* = 5) from The Jackson Laboratories (Bar Harbor, ME; strain B6C3-Tg(APPswe, PSEN1dE9)85Dbo/J). This particular mouse model corresponds to a form of early onset disease and expresses a mutant human presenilin 1 and a chimeric mouse/human amyloid precursor protein (APP_Swe_). The expression of both transgenes was directed by the mouse prion protein promoter. The APPswePS1 strain was developed on a B6C3HF2 background. The chimeric APP was modified to encode the Sweedish mutations K595N/M596L in order to elevate the amount ot A*β* produced from the transgene, by favoring processing through the beta-secretase pathway. Mice with the double mutation (APP/PS1) were generously donated (*N* = 33) by Dr. David Russell (UT Southwestern). Mice with the triple mutation (APP/PS1/Tau) were generated (*N* = 2) by Dr. Malu Tansey (UT Southwestern), to express the knock-in human presenilin 1 mutation, mutant Tau, and the Swedish APP mutation.

## 3. Statistical Analysis

Data were entered into Excel worksheets (Microsoft Corporation, Redmond, WA), and analyzed using the non-paired, two-tailed Student's *t*-test with unequal variance. *P* < .05 was regarded as significant. 

## 4. Results

### 4.1. In Vitro Binding of ^125^I-CQ to Brain Tissue

**Figure 1:**
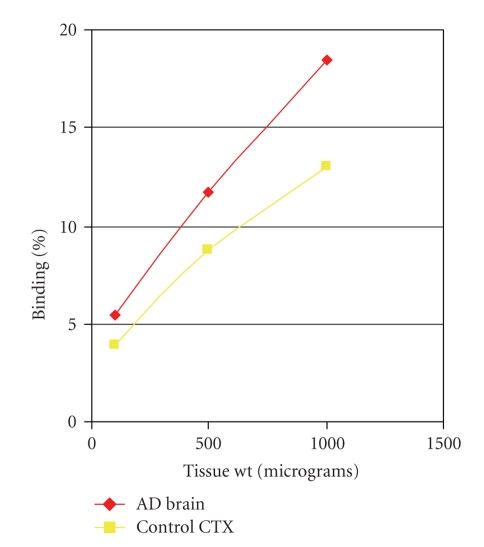
In vitro binding of ^125^I-CQ to brain homogenates.

^125^ICQ was used in in vitro assays of human postmortem frontal cortex to test the affinity of the radiolabelled chelator for amyloid plaques. Age-matched normal specimens were tested as control samples; no AD pathology was found in normal tissue (i.e., plaques and tangles in the brain). Experimental results (Figure 1) provide evidence of preferential binding by ^125^I-CQ to the AD brain tissue (1000 *μ*g, 18.5% binding), as compared to cortical control brain tissue (1000 *μ*g, 13% binding). Therefore, an amyloid-affinity drug could be successfully radiolabelled; the radioligand discriminated between AD brain tissue and control brain tissue. 

### 4.2. In Vivo Biodistribution of ^125^I-CQ BCA NPs

**Figure 2:**
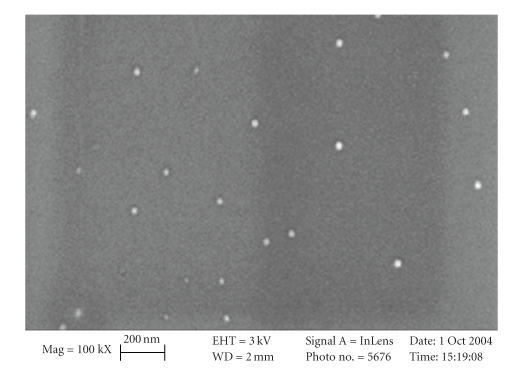
PBCA Nanoparticles.

The BCA monomer was polymerized with small particulate diameter (45 nm) and with uniform size distribution (Figure 2). In vivo biodistribution experiments showed that the free ^125^I-CQ had a rapid brain uptake, as well as rapid blood clearance in normal mice (Table 1). Furthermore, ^125^I-CQ cleared the brain quickly; the % ID/g for the brain at 2 minutes was 0.99 ± 0.40%, and at 4 hours, it was 0.04 ± 0.02%. Table 2 shows that when the ^125^I-CQ was encapsulated within the BCA NPs, the brain uptake was enhanced. At two minutes, the wild type mice exhibited 2.31 ± 0.89% uptake of the ^125^I-CQ BCA NPs in the brain (Table 1). Brain and blood clearances of the ^125^I-CQ BCA NPs were rapid; the % ID/g (brain) at 4 hours was 0.02%. Together, Tables 1 and 2 show that ^125^I-CQ BCA NPs have an increased brain uptake versus ^125^I-CQ in the wild type control mouse. 

Table 3 shows similar results, in Dynamic Light Units/mm^2^ (DLU/mm^2^), the unit described in autoradiographic imaging. For example, in the wildtype control mouse, at 10 minutes post injection the brain uptake of encapsulated ^125^I-CQ is 1.64 × 10^5^ ± 1.75 × 10^4^ versus 2.26 × 10^5^ ± 4.22 × 10^5^ in the AD transgenic mouse. Moreover, the brain uptake is significantly greater in the AD mouse at 90 minutes post injection of ^125^I-CQ BCA NPs (1.98 × 10^5^ ± 2.22 × 10^4^ wildtype versus 2.54 × 10^5^ ± 5.31 × 10^4^ AD transgenic)

Triple transgenic mice, for which mutations in the APP and Tau genes result in amyloid plaques and neurofibrillary tangles, were tested against wildtype controls for brain uptake of ^125^I-CQ BCA NPs. The in vivo uptake was imaged by autoradiography, and the data are given (DLU/mm^2^) in Table 4. Table 4 shows that at both 60 minutes and 90 minutes post injection of ^125^I-CQ BCA NPs, the AD transgenic mouse had a significantly greater brain uptake and retention of the imaging agent, as compared with the wildtype control. For example, at 60 minutes post injection, the brain uptake in the AD mouse was 6.28 × 10^6^ ± 1.68 × 10^6^ versus 3.92 × 10^6^ ± 1.49 × 10^6^ in the wildtype control. 

### 4.3. In Vivo Imaging of the Amyloid Plaques in AD Mouse Models

The PBCA NPs were successfully loaded with the radiolabelled quinoline derivative ^125^I-CQ and delivered to the mice by intravenous administration. Storage Phosphor imaging qualitatively showed that the NPs transported the drug across the BBB. Further, the ^125^I-CQ labeled the amyloid deposits. 

**Figure 3:**
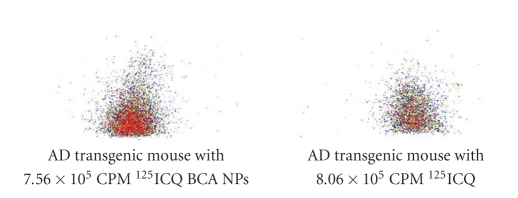
AD transgenic mice injected with ^125^ICQ BCA NPs and ^125^ICQ (Mice aged 7 mo; 15 minutes post injection).

**Figure 4:**
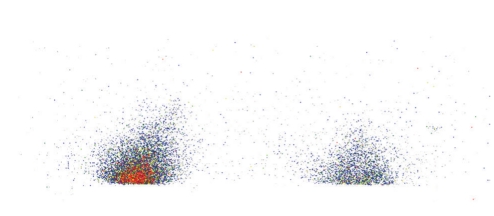
AD transgenic mice injected with ^125^ICQ BCA NPs and ^125^ICQ (Mice aged 7 mo; 90 minutes post injection).

**Figure 5:**
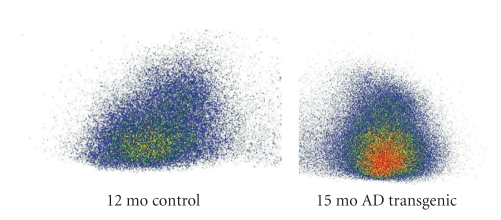
^125^ICQ BCA NPs (1 hour postinjection).

Figure 3 shows in vivo autoradiographs of the brain uptakes of ^125^ICQ BCA NPs and ^125^ICQ in transgenic mice (7 months old, APP/PS1), at 15 minutes postadministration of the nanoparticle-encapsulated drug and the free drug. The AD transgenic mice have a greater brain uptake with the use of nanoparticles, as compared to the free ^125^ICQ; thus, nanoparticulate encapsulation of ^125^ICQ enhances BBB crossing of the drug. Furthermore, nanoparticulate encapsulation of ^125^ICQ enhances retention of the drug. Figure 4 shows in vivo autoradiographs of the brain uptakes of ^125^ICQ BCA NPs and ^125^ICQ in transgenic mice (7 months old, APP/PS1^⋆^), at 90 minutes post administration of the nanoparticle-encapsulated drug and the free drug. At 90 minutes post injection, the AD transgenic mice have a greater brain uptake with the nanoparticles, as compared to the free ^125^ICQ. Therefore, transgenic mice have longer brain retention of the nanoparticle delivered drug, as compared to the free drug. Figure 5 shows in vivo autoradiographs of the brain uptakes of ^125^ICQ BCA NPs in a 12-month wild type control mouse, and in a 15-month AD transgenic mouse (APP/PS1^⋆^). At one hour post injection, the AD transgenic mouse has an increased brain uptake of the nanoparticles, presumably due to the presence of amyloid plaques.

**Figure 6:**
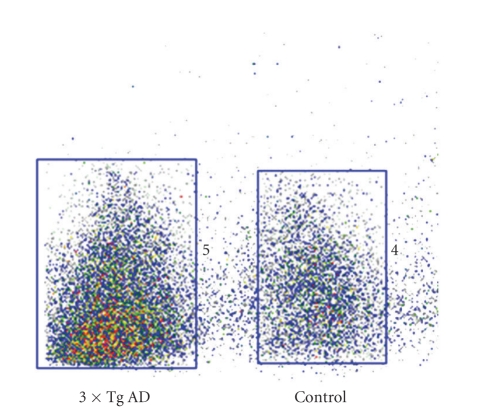
^125^ICQ BCA NPs triple transgenic mouse model (mice aged 12 mo; 1 hour postinjection).

**Figure 7:**
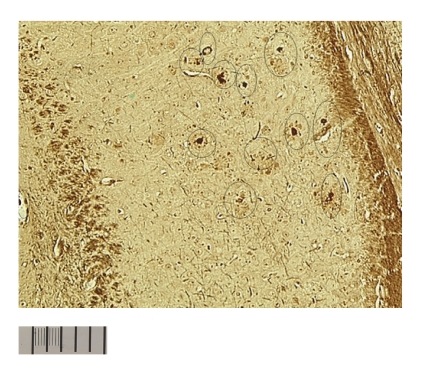
Hippocampal pathology in 3 × Tg Bielschowsky Stain.

**Figure 8:**
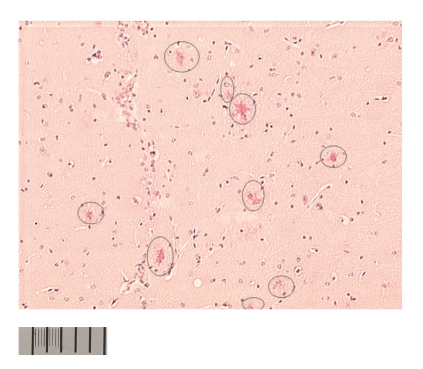
Aggregated amyloid plaques: 15 mo AD transgenic mouse.

**Figure 9:**
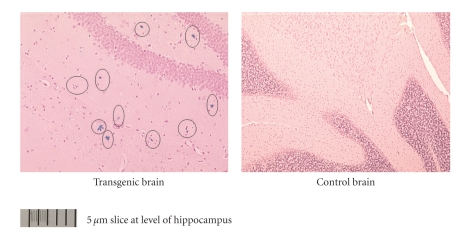
15 mo AD Transgenic: hippocampal staining of Fe^2+^.

**Figure 10:**
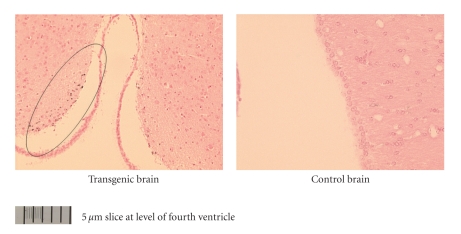
Cu^2+^ staining of aggregates in 3 × Tg (APP/PS1/Tau) mouse.

**Figure 11:**
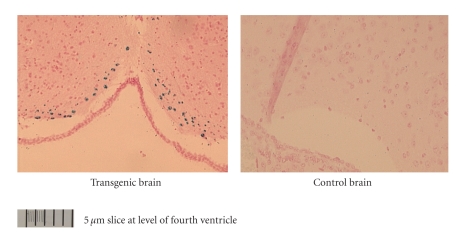
Fe^2+^ staining in 3 × Tg mouse.

Experiments with ^125^ICQ BCA NPs were also done in AD triple transgenic (3 × Tg) mice (APP/PS1/Tau). In Figure 6, in vivo autoradiographs show that at one hour post injection, the AD triple Transgenic mouse had the higher brain uptake of the nanoparticles (as compared to the wild type control mouse). Additionally, a Bielchowsky stain (Figure 7) verified the AD pathology in the 3 x Tg mouse. Finally, histological staining of brain slices taken from these AD mouse models verified the presence of amyloid (Congo red, Figure 8), Fe^2+^ (Prussian blue, Figures 9 and 11), and Cu^2+^ (Rubeanic acid, Figure 10).

## 5. Discussion

Cyanoacrylate nanoparticles were specifically designed for carrying drugs across the blood-brain barrier in mouse models of Alzheimer's disease. In vivo biodistribution of the ^125^I-CQ-labelled butylcyanoacrylates in wild-type mice showed that they crossed the BBB with greater efficiency than the ^125^ICQ control. The BCA NP was selected as the prototype drug carrier because it polymerized with the most reproducibility; it crossed the BBB and had a rapid uptake and clearance from the normal brain. These parameters are important in order to validate the PBCA NP as a drug carrier across the BBB; another unique factor is the lipophilicity of the carrier. Good lipophilicity assists rapid uptake and clearance from the normal brain [17]. The lipophilicity of the nanoparticles was enhanced by surfactant coating with Tween-80. 

The prototype PBCA NP was fully characterized for physicochemical and stabilizer effect. Smaller sized particles resulted when the monomer was polymerized at a lower pH, in the presence of the stabilizer Dextran 70 000 (as opposed to the hydrophilic polyethylene glycol, PEG), and definitely in the presence of a surfactant (e.g., Tween-80). Loading of the nanoparticles with amyloid-affinity drugs, such as derivatives of the Thioflavins (S- or T-), or Congo red did not significantly affect the size of the BCA NPs. Therefore, the BCA NPs maintained stability upon drug loading in vitro. Additional amyloid-affinity drugs have been reviewed in the literature [2, 17, 18]. 

The amyloid-affinity chelator CQ was successfully radioiodinated with ^125^I; ^125^ICQ was used in in vitro assays of human postmortem frontal cortex to test the affinity of the radiolabelled chelator for amyloid plaques. Autoradiography validated preferential labeling of the AD tissue by the ^125^ICQ (compared with control brain tissue). Then, the ^125^ICQ was successfully encapsulated within PBCA NPs. ^125^ICQ PBCA NPs preferentially labeled frontal human AD tissue compared with frontal control tissue. PBCA NPs act as drug carriers of ^125^ICQ targeted towards amyloid-beta plaques, presumably due to chelation of transitional metals in amyloid plaques. 

## 6. Conclusion

In vivo detection of amyloid plaques for the early diagnosis of AD is desirable. Presently, histological confirmation of the plaques and of the neurofibrillary tangles is the only definitive mode of diagnosis. This is true despite the fact that patients typically receive clinical diagnoses based on cognitive tests, medical histories, and so forth. Noninvasive in vivo detection affords patients the opportunity to receive the most effective patient care as early as possible. Likewise, it allows clinicians the prospect of tracking disease progression when definitive treatment becomes available. Hence, healthcare professionals are able to design appropriate therapeutic strategies. 

In vivo detection of amyloid-beta proteins improves specificity of diagnosis in noninvasive screening techniques, such as single photon emission computed tomography (SPECT) imaging. We have designed polybutylcyanoacrylate nanoparticles with incorporated radioligands and amyloid affinity agents that are attracted to the A*β* proteins. Thus, the clinical potential of the ^125^ICQ-BCA-NPs is improved specificity of diagnostic accuracy in AD detection. 

## Figures and Tables

**Table 1:** In vivo biodistribution of ^125^I-CQ by IV administration in wild type mice (*n* = 105) (table represents organ uptakes in %ID/g ± SD).

Time (min)	Brain	Blood	Spleen	Liver	Brain : blood ratio
2	1 ± 0.4	9.2 ± 2.8	2.6 ± 0.1	12.5 ± 0.8	0.1 ± 0.1
5	1 ± 0.3	5.7 ± 1.3	N/A	15.5 ± 5.6	0.2 ± 0.01
15	0.3 ± 0.1	2.9 ± 0.7	1.2 ± 0.1	3.8 ± 0.7	0.1 ± 0.1
30	0.3 ± 0.1	2.3 ± 0.4	1.3 ± 1	5.2 ± 2.2	0.1 ± 0.1
60	0.1 ± 0.1	1.3 ± 0.7	0.5 ± 0.3	3.6 ± 2.3	0.3 ± 0.5
120	0.03 ± 0.03	0.1 ± 0.1	0.6 ± 0.4	1.3 ± 0.5	0.2 ± 0.1
240	0.04 ± 0.02	0.6 ± 0.2	0.4 ± 0.3	0.9 ± 0.2	0.1 ± 0.1

*P* = .05

**Table 2:** In vivo biodistribution of ^125^I-CQ-BCA-NPs by IV administration in wild type mice (*n* = 105) (NP polymerized in the presence of 1% Dextran 70 000 and 1% Tween-80; the table represents organ uptakes in %ID/g ± SD).

Time (min)	Brain	Blood	Spleen	Liver	Brain:bloodratio
2	2.3 ± 0.9	12 ± 2.5	1.6 ± 0.4	15 ± 2.1	0.2 ± 0.1
5	1.5 ± 0.9	7.9 ± 1.6	1.8 ± 0.5	11.4 ± 1.5	0.2 ± 0.1
15	0.5 ± 0.2	4.9 ± 1.2	1.4 ± 0.5	8.1 ± 2.1	0.1 ± 0.1
30	0.3 ± 0.1	3.4 ± 1.5	0.9 ± 0.7	6.6 ± 2.4	0.1 ± 0.0
60	0.2 ± 0.1	2.1 ± 1.1	0.5 ± 0.3	3.9 ± 1.6	0.1 ± 0.0
120	0.03 ± 0.0	0.7 ± 0.2	0.2 ± 0.03	2.3 ± 0.4	0.1 ± 0.0
240	0.02 ± 0.0	0.4 ± 0.0	0.1 ± 0.1	1.4 ± 0.1	0.01 ± 0.0

*P* = .05

**Table 3:** 7 month AD transgenic with 9 month control with ^125^ICQ BCA NPs in DLU/mm^2^ (mean ± SD); (*n* = 4).

Time (mins)	9 mo control	7 mo AD transgenic
10	1.64 × 10^5^ ± 1.75 × 10^4^	2.26 × 10^5^ ± 4.22 × 10^5^
30	1.76 × 10^5^ ± 3.49 × 10^4^	1.63 × 10^5^ ± 2.44 × 10^4^
60	1.06 × 10^5^ ± 1.57 × 10^4^	7.04 × 10^4^ ± 1.14 × 10^4^
90	1.98 × 10^5^ ± 2.22 × 10^4^	2.54 × 10^5^ ± 5.31 × 10^4^

*P* = .05; 1 mg BCA NP; 795,338 CPM ^125^ICQ.

**Table 4:** 12 months triple transgenic Mice with ^125^ICQ BCA NPs in DLU/mm^2^ (mean ± SD); (*n* = 2).

Time (min)	Control	Triple transgenic
5	4.94 × 10^6^ ± 1.61 × 10^6^	4.60 × 10^6^ ± 1.22 × 10^6^
30	1.53 × 10^5^ ± 8.26 × 10^3^	1.77 × 10^5^ ± 1.33 × 10^4^
60	3.92 × 10^6^ ± 1.49 × 10^6^	6.28 × 10^6^ ± 1.68 × 10^6^
90	3.94 × 10^6^ ± 5.05 × 10^5^	4.85 × 10^6^ ± 1.00 × 10^5^

*P* = .05; 1 mg BCA NP; 2.24 x 10^6^ CPM ^125^ICQ.
